# Characteristics of falls occurring during rehabilitation in an acute care hospital in older and non-older patients: A retrospective cohort study

**DOI:** 10.3389/fmed.2022.969457

**Published:** 2022-08-17

**Authors:** Tokio Kinoshita, Yukihide Nishimura, Yasunori Umemoto, Shinji Kawasaki, Yoshinori Yasuoka, Kohei Minami, Yumi Koike, Fumihiro Tajima

**Affiliations:** ^1^Department of Rehabilitation Medicine, Wakayama Medical University, Wakayama, Japan; ^2^Department of Rehabilitation Medicine, Iwate Medical University, Morioka, Japan

**Keywords:** incident, accident, patient safety, inpatients, rehabilitation

## Abstract

**Introduction:**

Although falls are often reported in hospitals and are common in older individuals, no reports on falls during rehabilitation exist. This study evaluated patients with falls occurring during rehabilitation and identified the characteristics of older and non-older patients.

**Materials and methods:**

Our study retrospectively analyzed reports of falls occurring during rehabilitation at a university hospital from April 1, 2020 to March 31, 2022. The survey items included the number of falls in the hospital as a whole and during rehabilitation, age, gender, modified Rankin Scale (mRS) before admission and at the time of fall, functional independence measure (FIM) at admission, patient communication status at the time of fall, and whether a therapist was near the patient. Patients aged ≥ 65 were considered older; aged ≤ 64, non-older; and those with the same age, gender, and clinical department, randomly selected as non-falling patients.

**Results:**

Thirty-five falls occurred during rehabilitation (14 in the non-older and 21 in the older patients), significantly lower than the 945 for the entire hospital, without any significant difference between non-older and older patients. No significant differences in mRS before admission and FIM at admission were noted for both groups in comparison with the non-falling patient group. Furthermore, gender, mRS, FIM, good communication status, and presence of therapist near the patient were similar between non-older and older patients (non-older 71.4%, older 52.4%). Most falls were minor adverse events that did not require additional treatment.

**Conclusion:**

The rate of falls during rehabilitation was much lower than that during hospitalization, and many falls had minimal impact on the patient. It was also difficult to predict falls in daily life and communication situations, and there was no difference in characteristics between the older and non-older groups. Since more than half of the falls occurred during training with the therapist, it is necessary to reconsider the training content.

## Introduction

According to the World Health Organization’s definition, a fall is “an event which results in a person coming to rest inadvertently on the ground or floor or other lower level” (1). Falls cause varying degrees of injury, loss of confidence, and reluctance to move. Furthermore, patients who fall during hospitalization have longer average lengths of stay and may incur additional costs than those who do not (2). Older adults often have several risk factors that increase the likelihood of falls, including impaired sensory and motor function, impaired integration of these systems (3), adverse drug events, and musculoskeletal disorders such as gait dysfunction and balance problems (4). Additionally, older patients in geriatric or rehabilitation wards have a higher risk of falls than that in other age groups (5, 6). Hospital falls are the most common safety incidents affecting older individuals, frequently causing family complaints, including civil claims. Therefore, an important medical safety measure includes understanding the characteristics of patients who experience falls and taking steps to prevent them. Preventive measures in hospitals include patient education, clinician education, environmental adaptation, use of assistive devices, exercise, scrutiny of medications, optimal nutritional guidance, cognitive impairment management, measures to mitigate disability due to falls, and development of leadership systems (7–11).

While falls in stroke patients admitted to rehabilitation wards and in older patients admitted to rehabilitation facilities are reported (12–14), no detailed investigations of falls that occur during actual rehabilitation practices exist. The authors have experienced adverse event (AE)s during rehabilitation that resulted in femur fractures, requiring additional surgery and prolonging hospital stays (15), and are keenly aware of the need to develop fall prevention strategies.

This study evaluated falls occurring during rehabilitation and obtained detailed information to improve risk management strategies for future rehabilitation care and identify characteristics of older and non-older patients.

## Materials and methods

### Study setting

As of 2022, Wakayama Medical University hospital has 760 general beds (including ten Intensive Care Unit beds) and 40 psychiatric beds, serving 27 clinical departments and 28 central medical treatment sections. Rehabilitation begins upon request from the physicians in various departments to the department of rehabilitation. Physiatrists examine inpatients prior to rehabilitation and evaluate their diagnosis, disease state, and physical condition. Registered and skilled therapists then commence exercise therapy. Thus, rehabilitation therapies are performed based on a thorough clinical examination and in accordance with each patient’s condition (16, 17).

### Study design

In this retrospective cohort study, we analyzed AE reports submitted by the Department of Rehabilitation Medicine to the Medical Safety Promotion Department at our hospital between April 2020 and March 2022.

### Data collection methods and procedures

At our hospital, all staff are required to report an AE to their corresponding risk manager. An AE during rehabilitation includes any instance that has caused or may have caused further physical or psychological injury to the patient (15).

The survey items were the number and contents of adverse events during the survey period, number of falls in the entire hospital and during rehabilitation, the age of fall patients, gender, modified Rankin Scale (mRS) at admission and time of fall, functional independence measure (FIM) at the time of admission, good communication rate of the patient at the time of the fall, presence or absence of a nearby therapist, and main clinical department. In addition, patients with matching age, gender, and department, who had not experienced a fall, were randomly selected for comparison as non-falling patients, and the same data were collected. The mRS (defines six levels of disability) and FIM (basic indicator of the severity of disability) were evaluated as indicators of Activities of Daily Living (ADL) (18–22). The FIM consists of 18 items, with a motor subscale (13 items) and cognition subscale (5 items), each of which is assessed using a 7-point ordinal scale.

The degree of impact the AE had on the patient was determined by the Medical Safety Promotion Department (15). According to the National Coordination Council for Medication Error Reporting and Prevention index (23), impacts of AEs are categorized into nine levels, as follows: category A (no error); categories B to D (error but no harm); categories E to H (error and harm), and category I (error and death). For AEs in categories A to D, no additional treatment is required. Specifically, category B refers to an error occurring but not reaching the patient (an “error of omission” does reach the patient). While category C pertains to an error occurred that reaches the patient but does not cause harm, category D is an error that reaches the patient and requires monitoring to confirm that it did not harm the patient and/or required intervention to preclude harm. For AEs in category E, minor treatment is required; however, for those in category F, intensive treatments are required and/or extension of hospital stay is needed. If permanent disability and sequelae with no significant or with significant functional or cosmetic problems develop, severe AEs are defined as G or H, respectively. Categories A to D that do not require additional treatment are classified as minor AEs (15).

### Statistical analysis

Data were grouped by age ≥ 65 years and ≤ 64 years, defined as older in the author’s country (24). The values of the variables are given as numbers, mean ± standard deviation (SD), and median (75th–25th percentiles), where applicable. The older and non-older and fall and non-fall groups were compared using the unpaired *t*-test for age and time from hospitalization and surgery to fall occurrence; the Mann–Whitney *U* test was used to evaluate the mRS and FIM; the χ^2^-test was used to evaluate the incidence of falls and gender differences, good communication rate of the patient, and whether a therapist was present at the time of the fall; and the Fisher’s exact test explored the main clinical departments of fallen patients. Differences were considered statistically significant at *p* < 0.05, and statistical evaluations were performed using the Graph Pad Prism 6 software (Graph Pad Software Inc, San Diego, California).

### Ethical considerations

This study was conducted in accordance with the Declaration of Helsinki, and the protocol was approved by the relevant ethics review committee (No. 3529). No additional risks were posed to patients during the data collection and analysis, and all related information was protected. Additionally, information concerning this study was posted on the university website, and patients or their families and relatives were given the opportunity to opt-out. The ethics review committee waived the requirement for patients’ written informed consent due to the retrospective nature of the study.

## Results

**FIGURE 1:**
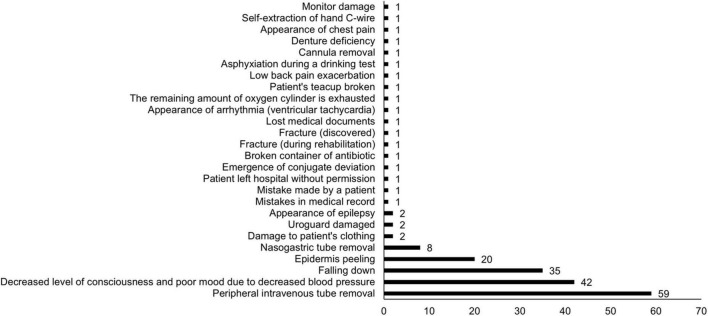
Adverse events that occurred during rehabilitation.

In the 2 years from April 2020, 46,050 patients were admitted to our hospital, of which 13,177 were rehabilitated. All AEs and occurrences during rehabilitation are shown in Figure 1. Of the 188 AEs that occurred during the 2-year period, falls were the third most common, following peripheral intravenous tube removal, and decreased level of consciousness and poor mood due to decreased blood pressure. Table 1 shows the number of falls that occurred during hospitalization and rehabilitation. The number of falls in the entire hospital was 945 and during rehabilitation was 35 (14 and 21 in the non-older and older groups, respectively), and the fall rate during rehabilitation was significantly lower (*p* < 0.0001) than that during hospitalization. While the incidence of falls during rehabilitation was 0.21 and 0.41% in the older and non-older patients, respectively, in older patients, it was about half that in the non-older patients; however, the difference was not significant (*p* = 0.0555).

**TABLE 1:** Number of falls occurring during hospitalization and rehabilitation.

	Number of falls	*p*-value
During hospitalization total (*n* = 46050)^a^	945 (2.05%)^c^	<0.0001^e^
During rehabilitation (*n* = 13177)^b^ Non-older (*n* = 3406)^b^ Older (*n* = 9771)^b^	35 (0.27%)^d^ 14 (0.41%)^d^ 21 (0.21%)^d^	0.0555^f^

^a^Is total number of hospitalized. ^b^Is number of patients referred to department of rehabilitation. ^c^Is number of falls/total number of hospitalized × 100. ^d^Is number of falls/number of patients referred to department of rehabilitation × 100. ^e^Is compares total inpatients to total falls during rehabilitation. ^f^Is comparison between non-older and older patients.

Table 2 shows the characteristics of the patients who fell grouped by age and matched with non-fall patients. There were no significant differences in mRS before admission and FIM at admission in both the non-older and older groups compared to the non-falling patients group. Furthermore, a comparison of non-older and older patients who fell showed no significant differences in mRS before admission, FIM at admission, days from admission to fall occurrence, days from surgery to fall occurrence, or mRS at fall.

**TABLE 2:** Characteristics of non-older and older adults who fell during rehabilitation.

	Non-older (≦64)				Older (≧65)			
	Total fall patients (*n* = 35)	Fall patients (*n* = 14)	Not-fall patients (*n* = 14)	*p*-value	Fall patients (*n* = 21)	Not-fall patients (*n* = 21)	*p*-value	Non-older vs. older
Age (years)	65.0 ± 16.8	49.6 ± 16.3	49.8 ± 16.4	0.9821	72.1 ± 5.7	72.1 ± 5.8	0.9790	*p* < 0.0001
Sex (female/male)	15/20	4/10	4/10	1	11/10	11/10	1	*p* = 0.1632
Pre-admission mRS (median IQR)	1 (0–2)	1 (0–2.75)	1 (0–2.5)	0.6252	1 (0–2)	1 (0–2)	>0.9999	*p* = 0.7397
FIM at admission (median IQR)	79 (46–115.5)	74 (39.0–99.25)	92 (48.25–120)	0.4265	93 (52–125)	98 (58–115)	0.9950	*p* = 0.3196
Motor FIM	47 (18.0–82.5)	41.5 (13–66.5)	64.5 (14.75–85)	0.3058	58 (23–90	55 (23–80)	0.9353	*p* = 0.2035
Cognition FIM	33 (25–35)	33 (24.75–34.75)	34.5 (24.25–36)	0.4459	31 (25–35)	34 (28–35)	0.4843	*p* = 0.8576
Days from hospitalization to occurrence	32.8 ± 31.5	39.4 ± 40.4			28.4 ± 23.2			*p* = 0.3216
Days from surgery to occurrence	26.1 ± 31.1	41.0 ± 47.3 (*n* = 7)			17.4 ± 8.7 (*n* = 12)			*p* = 0.1178
mRS at AE occurrence (median IQR)	4 (4–4)	4 (4–4)			4 (3–4)			*p* = 0.5785

mRS, modified Rankin Scale; IQR, 75th–25th percentiles; FIM, functional independence measure; AE, adverse event. The not-fall group was randomly selected from a group of patients matched by age, sex, and primary department.

Table 3 shows the details of the fall, patient’s communication status at the time of the fall, and whether the therapist was with the patient. Ten of 14 (71.4%) of the non-older and 11 of 21 (52.4%) of older patients fell despite good communication; however, there was no significant difference between the two (*p* = 0.2598). In addition, 10 of 14 (71.4%) non-older and 11 of 21 (52.4%) older patients fell even though the therapist was nearby (assisting), with no significant difference between the two (*p* = 0.2598). Furthermore, falls in both groups most commonly occurred while walking.

**TABLE 3:** About the communication ability of the patient at the time of the fall and details of the fall situation.

Non-older (*n* = 14)	Older (*n* = 21)
Good communication (*n* = 10)	Good communication (*n* = 11)
*The therapist was near the patient* *(n = 8)*	*The therapist was near the patient* *(n = 5)*
During stand-up training 1	During sitting training 1
During walking training 5	During walking training 4
Wheelchair-driven 1	*The therapist was not near the patient (n = 6)*
During transfer from bed to wheelchair 1	During stand-up training 1
*The therapist was not near the patient* *(n = 2)*	During walking training 1
During stand-up training alone 1	During walking alone 2
Legs touched the desk when moving 1	Legs touched the desk when moving 1
	Falling when doing exercises other than instructions 1

Poor communication (*n* = 4)	Poor communication (*n* = 10)
*The therapist was near the patient* *(n = 2)*	*The therapist was near the patient* *(n = 6)*
During walking training 1	During walking training 5
Falling out of a wheelchair 1	When standing from a wheelchair 1
*The therapist was not near the patient* *(n = 2)*	*The therapist was not near the patient* *(n = 4)*
During standing training 1	Resting in the sitting position 1
While climbing stairs alone 1	Standing up alone 2
	Falling out of a wheelchair 1

There was no significant difference between the two groups regarding the orientation status of non-older and older patients who fell and whether the therapist was near the patient. *p* = 0.2598 for both.

**FIGURE 2:**
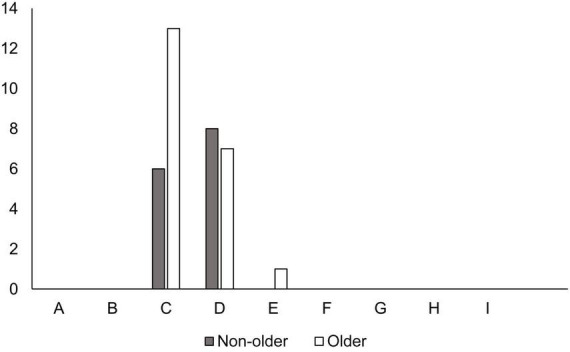
Impact of falls on patients. The x-axis is classification by National Coordination Council for Medication Error Reporting and Prevention index (reference 23), and the y-axis is number of falls.

The impact of falls on non-older patients was in categories C and D, where all fall patients did not require additional treatment. However, even in older patients, 20 out of 21 were in category C or D, with only one in category E in which a fall occurred during walking training while accompanied by a therapist and a wound was sutured due to an eyelid laceration (Figure 2). In the non-older group, more patients in the main departments of neurosurgery (28.6%), orthopedic surgery (14.3%), and rehabilitation (14.3%) had falls, while older patients also had more patient falls in neurosurgery (28.6%), orthopedic surgery (23.8%), and rehabilitation medicine (9.5%; Table 4). There was no significant difference in the main clinical departments of fallen patients between the non-older and older.

**TABLE 4:** Main clinical departments of fallen patients.

	Total patients (*n* = 35)	Non-older (*n* = 14)	Older (*n* = 21)	*p*-value
Neurosurgery	10 (28.6%)	4 (28.6%)	6 (28.6%)	>0.9999
Orthopedic surgery	7 (20.0%)	2 (14.3%)	5 (23.8%)	0.6760
Rehabilitation	4 (11.4%)	2 (14.3%)	2 (9.5%)	>0.9999
Diabetes, endocrine, and metabolic medicine	2 (5.7%)	1 (7.1%)	1 (4.8%)	>0.9999
Gastrointestinal, endocrine, and pediatric surgery	2 (5.7%)	1 (7.1%)	1 (4.8%)	>0.9999
Neuropsychiatry	2 (5.7%)	1 (7.1%)	1 (4.8%)	>0.9999
Cardiovascular medicine	1 (2.9%)	1 (7.1%)	0 (0%)	0.4000
Rheumatology and clinical immunology	1 (2.9%)	1 (7.1%)	0 (0%)	0.4000
Pediatrics	1 (2.9%)	1 (7.1%)	0 (0%)	0.4000
Emergency medicine	1 (2.9%)	0 (0%)	1 (4.8%)	0.4400
Cardiovascular, respiratory, and breast surgery	1 (2.9%)	0 (0%)	1 (4.8%)	0.4400
Plastic and reconstructive surgery	1 (2.9%)	0 (0%)	1 (4.8%)	0.4400
Nephrology (artificial dialysis)	1 (2.9%)	0 (0%)	1 (4.8%)	0.4400
Otolaryngology	1 (2.9%)	0 (0%)	1 (4.8%)	0.4400

## Discussion

In this study, the number of falls that occurred during rehabilitation was significantly lower than that during hospitalization and being older did not increase the number of falls. Furthermore, the ADL status before illness and at admission for both non-older and older patients was not related to the occurrence of falls. In addition, older patients with poor communication were not more likely to fall. This is the first report to investigate the characteristics related to patients who fall between non-older and older patients occurring during rehabilitation in an acute care hospital.

The risk of falls and injury increases with age and falls in older individuals are more likely to occur in association with social, behavioral, and physical risk factors, such as reduced physical fitness; impaired vision, balance, and gait; and multiple medications as well as physical environmental risk factors such as poor lighting and slippery floors (25, 26). People ≥ 65 years are at a high risk of falls, with 30% > 65 and 50% > 80 falling at least once a year (27). However, there was no significant difference in the incidence of falls in this study, and the actual incidence was 0.21% in older patients, lower than 0.41% in non-older patients. Lee et al. (13) reported that fall patients had a significantly lower total FIM score on admission than that of non-fall patients, and that patients with a low cognition FIM on admission had a higher risk of falls. Patients with Mini Mental State Examination (MMSE) scores < 28/30 had a nearly threefold increased risk of falls compared to control patients with 30/30 scores, indicating a relationship between cognitive ability and falls (28). Thirty-two patients who were unable to communicate their basic needs (21.7%) were almost twice as likely to fall during hospitalization as those who were able to communicate (29). Furthermore, Teasell et al. (30) reported that the group of patients who fell had significantly lower scores on the Berg Balance Scale and FIM than those from the non-fall group. Additionally, the number of falls significantly increased with lower FIM in the patients who fell. Pils et al. (31) also reported that being older, gender (higher fall rates in men), and MMSE scores were associated with falls during hospitalization for rehabilitation after femur fracture. In our study, there were no significant differences in mRS, motor FIM, cognition FIM before admission in the two groups, or in the ratio of mRS and good communication at the time of fall. Furthermore, there were no significant differences in either the older or non-older patients when compared to the matched non-fall group. Significantly, the incidence of falls during acute rehabilitation was not related to ADL performance or degree of communication as previously reported.

Schwendimann et al. (32) investigated 3,842 fall patients out of 34,972 inpatients, of which 2,552 (66.4%) were intact and 1,142 (29.7%) had minor injuries such as pain, bruise, blood type, and laceration. Additionally, 148 (3.9%) reported serious AEs such as fractures and intracranial hemorrhage. Of the 1,472 patients admitted to the rehabilitation center, 140 fell during their stay; 90% did not suffer harm, while 8/10 who did suffer harm had minor contusions, lacerations, or abrasions, and 2/10 suffered fractures (13). Saverino et al. (33) also reported 40/320 post-acute orthopedic and neurologic inpatients fell during hospitalization, with one sustaining a rib fracture; however, the falls otherwise had minor effects. The results of our study support previous findings that most falls do not have serious consequences, as 34/35 total falls were minor AEs that did not require additional treatment. However, follow-up studies of older adults who fell show that even non-injurious falls are associated with subsequent decline in basic and instrumental ADLs. Additionally, two or more non-injurious falls are associated with decreased social activity, and at least one injurious fall is associated with decreased physical activity (34). Therefore, attention to fall prevention is necessary regardless of the degree to which a fall affects the patient.

In this study, falls were more common in neurosurgical patients in both groups. In a one-year survey of falls in acute care hospitals (35), 826 of 49,059 inpatients experienced falls, with the most common primary diseases being neurological 214 (26%), gastroenterological 145 (18%), pediatrics 57 (7%), respiratory 51 (6%), cardiac 41 (5%), otolaryngology 40 (5%), orthopedics 33 (4%), and others 245 (30%). Eileen et al. (36) reported 3.38 falls per 1,000 patients per day in an academic hospital with 1,300 beds, with the highest rate of falls in neurology and internal medicine at 6.12. Stroke patients have a higher risk of experiencing falls due to multiple intrinsic risk factors, including impaired consciousness, cognitive impairment, ADL impairment, and depressive symptoms (37, 38). However, it is difficult to generalize as the disease severity of patients, severity of the disease to be treated at the study facility, number of patients accepted, and presence or absence of rehabilitation are unknown, more attention to fall prevention during rehabilitation for both older and non-older patients with cerebrovascular disorders should be paid, as in previous reports.

In previous studies, more than 80% falls in hospitals were not witnessed, and most falls occurred in situations where no one was nearby (9, 39). However, more than half of the falls during this study occurred in situations where the therapist was nearby (assisting) both the older and non-older groups. Patients offered rehabilitation in this hospital often have multiple serious motor, cognitive, and ADL impairments, and require a high amount of assistance. The difference in results as compared to previous studies may be related to the fact that the falls occurred during rehabilitation therapy sessions and that therefore the severity of the patient’s disability was different.

This study has the limitation of being a single-site, retrospective cohort study, which affects generalizations. Thus, it is important to conduct a larger, multi-site study in the future.

## Conclusion

The rate of falls during rehabilitation was significantly lower than that during hospitalization, and many falls had minimal impact on the patient. It was also difficult to predict falls by ADL and communication status, and there appeared to be no difference in characteristics between the older and non-older groups who experienced falls. However, since more than half of falls occur during training with a therapist near the patient, it is necessary to scrutinize the patient’s disability status and training content.

## Data availability statement

The original contributions presented in this study are included in the article/supplementary material, further inquiries can be directed to the corresponding author.

## Ethics statement

The studies involving human participants were reviewed and approved by the study was conducted according to the guidelines of the Declaration of Helsinki, and approved by the Ethics Review Committee of Wakayama Medical University. Written informed consent from the participants or their legal guardian/next of kin was not required to participate in this study in accordance with the national legislation and the institutional requirements.

## Author contributions

TK and YN conceptualized and designed the study, drafted, reviewed, and revised the manuscript. YU, YK, SK, YY, and KM designed the data collection instruments, collected data, performed the initial analyses, reviewed, and revised the manuscript. FT designed the data collection instruments, coordinated and supervised data collection, and critically reviewed the manuscript. All authors approved the final manuscript as submitted and agreed to be accountable for all aspects of the work.

## References

[1] World Health Organization [WHO]. *Falls.* Available online at: https://www.who.int/news-room/fact-sheets/detail/falls (accessed June 10, 2022).

[2] Morello RT Barker AL Watts JJ Haines T Zavarsek SS Hill KD The extra resource burden of in-hospital falls: a cost of falls study. *Med J Aust.* (2015) 203:367. 10.5694/mja15.00296 26510807

[3] Nitz JC Choy NL. The efficacy of a specific balance-strategy training programme for preventing falls among older people: a pilot randomised controlled trial. *Age Ageing.* (2004) 33:52–8. 10.1093/ageing/afh029 14695864

[4] de Jong MR Van der Elst M Hartholt KA. Drug-related falls in older patients: implicated drugs, consequences, and possible prevention strategies. *Ther Adv Drug Saf.* (2013) 4:147–54. 10.1177/2042098613486829 25114778 PMC4125318

[5] Haines TP Hill KD Bennell KL Osborne RH. Patient education to prevent falls in subacute care. *Clin Rehabil.* (2006) 20:970–9. 10.1177/0269215506070694 17065540

[6] Schwendimann R Bühler H De Geest S Milisen K. Characteristics of hospital inpatient falls across clinical departments. *Gerontology.* (2008) 54:342–8. 10.1159/000129954 18460867

[7] Cameron ID Dyer SM Panagoda CE Murray GR Hill KD Cumming RG Interventions for preventing falls in older people in care facilities and hospitals. *Cochrane Database Syst Rev.* (2018) 9:CD005465. 10.1002/14651858.CD005465.pub4 30191554 PMC6148705

[8] Oliver D Healey F Haines TP. Preventing falls and fall-related injuries in hospitals. *Clin Geriatr Med.* (2010) 26:645–92. 10.1016/j.cger.2010.06.005 20934615

[9] Hitcho EB Krauss MJ Birge S Claiborne Dunagan W Fischer I Johnson S Characteristics and circumstances of falls in a hospital setting: a prospective analysis. *J Gen Intern Med.* (2004) 19:732–9. 10.1111/j.1525-1497.2004.30387.x 15209586 PMC1492485

[10] Heng H Jazayeri D Shaw L Kiegaldie D Hill AM Morris ME. Hospital falls prevention with patient education: a scoping review. *BMC Geriatr.* (2020) 20:140. 10.1186/s12877-020-01515-w 32293298 PMC7161005

[11] Tricco AC Thomas SM Veroniki AA Hamid JS Cogo E Strifler L Comparisons of interventions for preventing falls in older adults: a systematic review and meta-analysis. *JAMA.* (2017) 318:1687–99. 10.1001/jama.2017.15006 29114830 PMC5818787

[12] Suzuki T Sonoda S Misawa K Saitoh E Shimizu Y Kotake T. Incidence and consequence of falls in inpatient rehabilitation of stroke patients. *Exp Aging Res.* (2005) 31:457–69. 10.1080/03610730500206881 16147463

[13] Lee JE Stokic DS. Risk factors for falls during inpatient rehabilitation. *Am J Phys Med Rehabil.* (2008) 87:341–50. 10.1097/PHM.0b013e31816ddc01 18427218

[14] Campanini I Mastrangelo S Bargellini A Bassoli A Bosi G Lombardi F Feasibility and predictive performance of the hendrich fall risk model II in a rehabilitation department: a prospective study. *BMC Health Serv Res.* (2018) 18:18. 10.1186/s12913-017-2815-x 29325560 PMC5765700

[15] Kinoshita T Kamijo YI Kouda K Yasuoka Y Nishimura Y Umemoto Y Evaluation of severe adverse events during rehabilitation for acute-phase patients: a retrospective cohort study. *Medicine.* (2022) 101:e29516. 35758395 10.1097/MD.0000000000029516 PMC9276444

[16] Kinoshita T Nishimura Y Nakamura T Hashizaki T Kojima D Kawanishi M Effects of physiatrist and registered therapist operating acute rehabilitation (PROr) in patients with stroke. *PLoS One.* (2017) 12:e0187099. 10.1371/journal.pone.0187099 29073250 PMC5658147

[17] Kinoshita T Yoshikawa T Nishimura Y Kamijo YI Arakawa H Nakamura T Mobilization within 24 hours of new-onset stroke enhances the rate of home discharge at 6-months follow-up: a prospective cohort study. *Int J Neurosci.* (2021) 131:1097–106. 10.1080/00207454.2020.1774578 32449874

[18] van Swieten JC Koudstaal PJ Visser MC Schouten HJ van Gijn J. Interobserver agreement for the assessment of handicap in stroke patients. *Stroke.* (1988) 19:604–7. 10.1161/01.str.19.5.604 3363593

[19] de Haan R Limburg M Bossuyt P van der Meulen J Aaronson N. The clinical meaning of Rankin “handicap” grades after stroke. *Stroke.* (1995) 26:2027–30. 10.1161/01.str.26.11.2027 7482643

[20] Heinemann AW Linacre JM Wright BD Hamilton BB Granger C. Relationships between impairment and physical disability as measured by the functional independence measure. *Arch Phys Med Rehabil.* (1993) 74:566–73. 10.1016/0003-9993(93)90153-2 8503745

[21] Linacre JM Heinemann AW Wright BD Granger CV Hamilton BB. The structure and stability of the functional independence measure. *Arch Phys Med Rehabil.* (1994) 75:127–32. 8311667

[22] Chumney D Nollinger K Shesko K Skop K Spencer M Newton RA. Ability of functional independence measure to accurately predict functional outcome of stroke-specific population: systematic review. *J Rehabil Res Dev.* (2010) 47:17–29. 10.1682/jrrd.2009.08.0140 20437324

[23] National Coordinating Council for Medication Error Reporting and Prevention. *NCC MERP Index for Categorizing Medication Errors.* (2001). Available online at: https://www.nccmerp.org/sites/default/files/indexBW2001-06-12.pdf (accessed June 10, 2022). 10.1002/pds.1423 17523185

[24] Ministry of Health, Labour and Welfare. *e-Health Net.* Available online at: https://www.e-healthnet.mhlw.go.jp/information/dictionary/alcohol/ya-032.html (accessed June 10, 2022).

[25] Ambrose AF Paul G Hausdorff JM. Risk factors for falls among older adults: a review of the literature. *Maturitas.* (2013) 75:51–61. 10.1016/j.maturitas.2013.02.009 23523272

[26] Feldman F Chaudhury H. Falls and the physical environment: a review and a new multifactorial falls-risk conceptual framework. *Can J Occup Ther.* (2008) 75:82–95. 10.1177/000841740807500204 18510252

[27] Swift CG Iliffe S. Assessment and prevention of falls in older people – concise guidance. *Clin Med.* (2014) 14:658–62. 10.7861/clinmedicine.14-6-658 25468853 PMC4954140

[28] Gleason CE Gangnon RE Fischer BL Mahoney JE. Increased risk for falling associated with subtle cognitive impairment: secondary analysis of a randomized clinical trial. *Dement Geriatr Cogn Disord.* (2009) 27:557–63. 10.1159/000228257 19602883 PMC2742559

[29] Sullivan R Harding K. Do patients with severe poststroke communication difficulties have a higher incidence of falls during inpatient rehabilitation? A retrospective cohort study. *Top Stroke Rehabil.* (2019) 26:288–93. 10.1080/10749357.2019.1591689 30890038

[30] Teasell R McRae M Foley N Bhardwaj A. The incidence and consequences of falls in stroke patients during inpatient rehabilitation: factors associated with high risk. *Arch Phys Med Rehabil.* (2002) 83:329–33. 10.1053/apmr.2002.29623 11887112

[31] Pils K Neumann F Meisner W Schano W Vavrovsky G Van der Cammen TJ. Predictors of falls in elderly people during rehabilitation after hip fracture – who is at risk of a second one?. *Z Gerontol Geriatr.* (2003) 36:16–22. 10.1007/s00391-003-0142-9 12616403

[32] Schwendimann R Bühler H De Geest S Milisen K. Falls and consequent injuries in hospitalized patients: effects of an interdisciplinary falls prevention program. *BMC Health Serv Res.* (2006) 6:69. 10.1186/1472-6963-6-69 16759386 PMC1534028

[33] Saverino A Benevolo E Ottonello M Zsirai E Sessarego P. Falls in a rehabilitation setting: functional independence and fall risk. *Eura Medicophys.* (2006) 42:179–84. 17039213

[34] Tinetti ME Williams CS. The effect of falls and fall injuries on functioning in community-dwelling older persons. *J Gerontol A Biol Sci Med Sci.* (1998) 53:M112–9. 10.1093/gerona/53a.2.m112 9520917

[35] Kobayashi K Imagama S Inagaki Y Suzuki Y Ando K Nishida Y Incidence and characteristics of accidental falls in hospitalizations. *Nagoya J Med Sci.* (2017) 79:291–8. 10.18999/nagjms.79.3.291 28878434 PMC5577015

[36] Aranda-Gallardo M Morales-Asencio JM Canca-Sanchez JC Toribio-Montero JC. Circumstances and causes of falls by patients at a Spanish acute care hospital. *J Eval Clin Pract.* (2014) 20:631–7. 10.1111/jep.12187 24902772

[37] Rapp K Ravindren J Becker C Lindemann U Jaensch A Klenk J. Fall risk as a function of time after admission to sub-acute geriatric hospital units. *BMC Geriatr.* (2016) 16:173. 10.1186/s12877-016-0346-7 27717326 PMC5054540

[38] Jørgensen L Engstad T Jacobsen BK. Higher incidence of falls in long-term stroke survivors than in population controls: depressive symptoms predict falls after stroke. *Stroke.* (2002) 33:542–7. 10.1161/hs0202.102375 11823667

[39] Hill AM Hoffmann T Hill K Oliver D Beer C McPhail S Measuring falls events in acute hospitals-a comparison of three reporting methods to identify missing data in the hospital reporting system. *J Am Geriatr Soc.* (2010) 58:1347–52. 10.1111/j.1532-5415.2010.02856.x 20487077

